# The bi-periodic Horadam sequence and some perturbed tridiagonal 2-Toeplitz matrices: A unified approach

**DOI:** 10.1016/j.heliyon.2022.e08863

**Published:** 2022-02-02

**Authors:** Milica Anđelić, Carlos M. da Fonseca, Fatih Yılmaz

**Affiliations:** a Department of Mathematics, Kuwait University, Safat 13060, Kuwait; b Kuwait College of Science and Technology, Doha District, Safat 13133, Kuwait; c Chair of Computational Mathematics, University of Deusto, 48007 Bilbao, Spain; d Department of Mathematics, Ankara Hacı Bayram Veli University, Turkey

**Keywords:** Horadam sequence, Chebyshev polynomials of second kind, Determinant, Tridiagonal matrices

## Abstract

In this note we consider the so-called bi-periodic Horadam sequences. Explicit formulas in terms of Chebyshev polynomials of the second kind and the determinant of some perturbed tridiagonal 2-Toeplitz matrices are established. Several illustrative examples are provided as well.

## 1 The Horadam sequence

In 1965, Alwyn F. Horadam considered the sequence $\left\{w_{n}\equiv w_{n}(a,b;p,q)\right\}$ defined by the second-order homogeneous linear recurrence

(1.1) $$w_{n}=pw_{n-1}-qw_{n-2}\;,\;\text{for }n⩾2\text{,}$$

with initial conditions

(1.2) $$w_{0}=a\;\text{and}\;w_{1}=b\;,$$

for arbitrary integers *a* and *b* [24, Section 3]. This is one of the possible extensions of the Fibonacci numbers, setting a=0 and b=p=−q=1. Horadam studied many of its properties and other instances [21], [22], [23]. If today $\left\{w_{n}\right\}$ is familiar, in the 1960's it was a great novelty. According to A.G. Shannon [31], [32],

*These generalizations were not only elegant, but they paved the way for clarifying the roles of the fundamental and primordial sequences introduced more than eighty years previously by [Édouard] Lucas. They stimulated work on other second and higher order recursive sequences, and the introduction of many techniques from the special functions of mathematical physics.*

Therefore, $\left\{w_{n}\equiv w_{n}(a,b;p,q)\right\}$ is commonly called *Horadam sequence*.

The Horadam sequence has been scrutinized for the past decades with many new extensions emerging in the literature. As an example, the reader is referred to [2] and the references therein.

On the other hand, the connection between particular cases of the Horadam sequence and the Chebyshev polynomials of the first and second kinds, has been a topic of discussion since the very early 1960's. Indeed, in [7], R.G. Buschman explores it, namely for the Fibonacci numbers, starting from the equality

(1.3) $$F_{n+1}={\left(-i\right)}^{n}\;U_{n}\left(\frac{i}{2}\right)\;,$$

where $F_{n}$ is the *n*th Fibonacci number and i represents the unit imaginary number. Recall the Chebyshev polynomials of the second kind ${\left\{U_{n}(x)\right\}}_{n⩾0}$ are the orthogonal polynomials satisfying the three-term recurrence relations

(1.4) $$U_{n+1}(x)-2xU_{n}(x)+U_{n-1}(x)=0\;,\;\text{for }\;n=0,1,2,\ldots \;,$$

with initial conditions $U_{-1}(x)=0$ and $U_{0}(x)=1$. One of the most well-known explicit formulas for these polynomials is

$$U_{n}(x)=\frac{\sin(n+1)\theta }{\sin\theta }\;,\;\text{with}\;x=\cos\theta \;(0⩽\theta <\pi ),$$

for all n=0,1,2….

It is important to note that, in general, setting

(1.5) $$T_{n}={\left(\begin{array}{cccc} p\; & 1\; & \; & \\ q\; & \ddots \; & \ddots \; & \\ \; & \ddots \; & \ddots \; & 1\\ \; & \; & q\; & p \end{array}\right)}_{n\times n},$$

we have (cf. e.g. [1], [14])

$$\det T_{n}={\left(\sqrt{q}\right)}^{n}\;U_{n}\left(\frac{p}{2\sqrt{q}}\right)\;.$$

As far as we know, the first explicit formula to the Horadam sequence in terms of Chebyshev polynomials of the second kind is due to Udrea [36]:

(1.6) $$w_{n}={\left(\sqrt{q}\right)}^{n}\;\left(\frac{b}{\sqrt{q}}\;U_{n-1}\left(\frac{p}{2\sqrt{q}}\right)-a\;U_{n-2}\left(\frac{p}{2\sqrt{q}}\right)\right)\;.$$

Originally, (1.6) is proved for n⩾2, but of course we can state for any nonnegative integer number *n*, provided $U_{-1}(x)=0$ and $U_{-2}(x)=-1$. Udrea's approach is based on the method for solving second order linear homogeneous recurrence relations and some trigonometric manipulations.

In this note, we proposed an approach based on elementary matrix theory. This will allow us find the solutions for recent extensions of the Horadam sequence.

In the next section, we will see how by perturbing some entries of the matrix $T_{n}$ defined in (1.5), one can provide an explicit formula for the Horadam sequence (1.1)–(1.2), thus obtaining (1.6). As a particular case, one can provide the solutions to recent problems in a more general context. In Section 4, we fully extend the recent results on the so-called generalized bi-periodic Fibonacci and Lucas sequences. Several examples are discussed. Clearly, our method can be extended to any type of periodicity.

## 2 Certain perturbed tridiagonal Toeplitz matrices

The perturbation of extreme entries of a Toeplitz tridiagonal matrix is a common long-standing subject of study in many areas of mathematics and its applications (cf. e.g. [8], [15], [16], [26]). We can also establish a close connection to the Horadam sequence. For example, if a=0, then

(2.1) $$w_{n}={\left|\begin{array}{ccccc} 0\; & 1\; & \; & \; & \\ -b\; & p\; & 1\; & \; & \\ \; & q\; & \ddots \; & \ddots \; & \\ \; & \; & \ddots \; & \ddots \; & 1\\ \; & \; & \; & q\; & p \end{array}\right|}_{\left(n+1)\times (n+1\right)}=b\;\det T_{n-1}=b\;{\left(\sqrt{q}\right)}^{n-1}\;U_{n-1}\left(\frac{p}{2\sqrt{q}}\right)$$

As a classical example, we have the Pell numbers, $P_{n}$, where a=0, b=1, p=2, q=−1. Consequently, we get the well-known formula $P_{n}={\left(-i\right)}^{n-1}U_{n-1}\left(i\right)$. Of course, another example is the identity (1.3).

Here our aim is to produce two perturbations in the first two main diagonal entries, namely,

(2.2) $${\overset{˜}{T}}_{n}={\left(\begin{array}{cccccc} p+r\; & 1\; & \; & \; & \; & \\ q\; & p+s\; & 1\; & \; & \; & \\ \; & q\; & p\; & 1\; & \; & \\ \; & \; & q\; & \ddots \; & \ddots \; & \\ \; & \; & \; & \ddots \; & \ddots \; & 1\\ \; & \; & \; & \; & q\; & p \end{array}\right)}_{n\times n}.$$

In this case, using the multilinearity of the determinant, we have

(2.3) $$\det{\overset{˜}{T}}_{n}=\det T_{n}+r\det T_{n-1}+s(r+p)\det T_{n-2}\;,$$

or, in terms of the Chebyshev polynomials of the second kind,

(2.4) $$\det{\overset{˜}{T}}_{n}={\left(\sqrt{q}\right)}^{n-2}(q\;U_{n}\left(\frac{p}{2\sqrt{q}}\right)+r\sqrt{q}\;U_{n-1}\left(\frac{p}{2\sqrt{q}}\right)+s(r+p)\;U_{n-2}\left(\frac{p}{2\sqrt{q}}\right)).$$

Assuming a≠0 means that, if we set

$$p+r=a\;\text{and}\;\left|\begin{array}{cc} a\; & 1\\ q\; & p+s \end{array}\right|=b$$

i.e.,

(2.5) $$r=a-p\;\text{and}\;s=\frac{b-ap+q}{a}\;,$$

then, from (2.4),

(2.6) $$\det{\overset{˜}{T}}_{n}={\left(\sqrt{q}\right)}^{n-1}\left(a\sqrt{q}\;U_{n}\left(\frac{p}{2\sqrt{q}}\right)+(b-ap)\;U_{n-1}\left(\frac{p}{2\sqrt{q}}\right)\right)\;.$$

and, therefore,

$$w_{n}=\det{\overset{˜}{T}}_{n+1}\;,\;\text{for any }n⩾0.$$

From (1.4) and (2.6) we obtain immediately (1.6). On the other hand, we notice that (2.6), for a=0, contains (2.1).

A derivation of the Pell numbers is the so-called Lucas-Pell numbers, $LP_{n}$, satisfying a=b=2, also known as companion Pell numbers. In this case, we have r=0 and $s=-\frac{3}{2}$. Then,

$$LP_{n}=i^{n-1}\left(-U_{n+1}\left(\frac{1}{i}\right)-3U_{n-1}\left(\frac{1}{i}\right)\right)={\left(-i\right)}^{n+1}\left(U_{n+1}\left(i\right)+3U_{n-1}\left(i\right)\right),$$

or

$$LP_{n}={\left|\begin{array}{cccccc} 2\; & 1\; & \; & \; & \; & \\ -1\; & \frac{1}{2}\; & 1\; & \; & \; & \\ \; & -1\; & 2\; & 1\; & \; & \\ \; & \; & -1\; & \ddots \; & \ddots \; & \\ \; & \; & \; & \ddots \; & \ddots \; & 1\\ \; & \; & \; & \; & -1\; & 2 \end{array}\right|}_{\left(n+1)\times (n+1\right)}.$$

In conclusion, any Horadam sequence (1.1) falls into (2.6) and can be interpreted as the determinant of a tridiagonal matrix.

## 3 An application

As an application of the previous section, we analyze a particular case of (1.1) considered recently by da Silva in [33]. There, it was defined the linear recurrence relation

(3.1) $$x_{n+2}=\frac{u}{v}x_{n+1}-x_{n}\;,\;\text{with }n⩾0\text{,}$$

where the initial conditions are arbitrary constants *a* and *b*, and proposed the solution

(3.2) $$x_{n}=\left(\begin{array}{cc} U_{n}\left(\frac{u}{2v}\right)\; & U_{n+1}\left(\frac{u}{2v}\right) \end{array}\right)\left(\begin{array}{cc} 1-\frac{u^{2}}{v^{2}}\; & \frac{u}{v}\\ \frac{u}{v}\; & -1 \end{array}\right)\left(\begin{array}{c} a\\ b \end{array}\right)\;,\;\text{with }n⩾0,$$

provided

(3.3) u≠±2v.

In this brief section, we present the solution for the linear recurrence relation (3.1) as an immediate consequence of (2.6).

For, set $p=\frac{u}{v}$ and q=1. Then, from (2.6) one gets

$$x_{n}=a\;U_{n+1}\left(\frac{u}{2v}\right)+\left(\frac{a^{2}+b^{2}}{b}\right)U_{n}\left(\frac{u}{2v}\right)\;.$$

Notice that, in order to prove (3.2), we need to impose the restriction (3.3). However, in our approach, only elementary linear algebra is required and this condition is redundant.

## 4 The bi-periodic Horadam sequence

Recently, it was considered in [9], [35] the generalization of the Horadam sequence defined by the recurrence relations

$${\overset{˜}{w}}_{n}=\left\{ \begin{array}{cc} p_{1}{\overset{˜}{w}}_{n-1}-q{\overset{˜}{w}}_{n-2}\; & \text{ if}\;\text{n}\;\text{is odd}\\ p_{2}{\overset{˜}{w}}_{n-1}-q{\overset{˜}{w}}_{n-2}\; & \text{ if}\;\text{n}\;\text{is even} \end{array}\right.$$

with arbitrary initial conditions ${\overset{˜}{w}}_{0}=a$ and ${\overset{˜}{w}}_{1}=b$. A special attention was given to ${\overset{˜}{w}}_{0}=0,{\overset{˜}{w}}_{1}=1$ and ${\overset{˜}{w}}_{0}=2,{\overset{˜}{w}}_{1}=b$, with several relations being established. Contrary to a common belief, the study of this recurrence relation goes back 1940's with the seminal paper [29], where periodic tridiagonal Toeplitz matrices were considered (cf. also [11]).

In the spirit of Section 2 and [2], [5], the aim of this section is to discuss the generalization of the Horadam sequence defined by the recurrence relation

(4.1) $$w_{n}^{\left(2\right)}=\left\{ \begin{array}{cc} p_{1}w_{n-1}^{\left(2\right)}-q_{2}w_{n-2}^{\left(2\right)}\; & \text{ if}\;\text{n}\;\text{is even}\\ p_{2}w_{n-1}^{\left(2\right)}-q_{1}w_{n-2}^{\left(2\right)}\; & \text{ if}\;\text{n}\;\text{is odd} \end{array}\right.$$

for arbitrary initial conditions $w_{0}^{\left(2\right)}=a$ and $w_{1}^{\left(2\right)}=b$. Clearly, $\left\{w_{n}^{\left(2\right)}\right\}$ is an extension of $\left\{{\overset{˜}{w}}_{n}\right\}$.

It is unclear the origin of the recurrence relation (4.1). In fact, it has been rediscovered independently in many instances and different areas of research several times over the last decades. In the most of the cases, the authors were unaware of the previous work. In 1978, Ferguson considers in [13] not only the (4.1) with initial conditions $w_{0}^{\left(2\right)}=0$ and $w_{1}^{\left(2\right)}=p_{1}$, but also the general case when the periodicity is greater than 2. This was largely motivated by Lehmer's paper [25] on the permanents of certain tridiagonal matrices. We shall be back to these matrices later on. Surprisingly, Lehmer's paper contains no citations.

As previously, our approach is based on the determinant of certain perturbed Toeplitz tridiagonal matrices.

The matrices of the form

(4.2) $$T_{n}^{\left(2\right)}={\left(\begin{array}{ccccccc} p_{1}\; & 1\; & \; & \; & \; & \; & \\ q_{1}\; & p_{2}\; & 1\; & \; & \; & \; & \\ \; & q_{2}\; & p_{1}\; & 1\; & \; & \; & \\ \; & \; & q_{1}\; & p_{2}\; & 1\; & \; & \\ \; & \; & \; & q_{2}\; & \ddots \; & \ddots \; & \\ \; & \; & \; & \; & \ddots \; & \ddots \; & \ddots \\ \; & \; & \; & \; & \; & \ddots \; & \end{array}\right)}_{n\times n}\;,$$

i.e., tridiagonal matrices $A_{n}=(a_{ij})$ with entries satisfying

$$a_{i+2,j+2}=a_{ij}\;,\;\text{for }i,j=1,2,\ldots ,n-2\;,$$

are known as *tridiagonal* 2*-Toeplitz matrices* [18], [19]. If instead of a period 2, we have a generic period *k*, these matrices are called *tridiagonal k-Toeplitz* matrices [17] whose determinant is known as *periodic continuant* [28]. As mentioned above, the study of these matrices goes back to 1947 Rutherford's pioneering paper [29]. Their determinant and spectral properties were studied independently in distinct contexts and applications (cf. [3], [4], [5], [6], [10], [11], [12], [17], [18], [19], [27]).

If $q_{1}=q_{2}=q$, then it is not difficult to see that from [29] (cf. also [11, pp. 32-33]), we get

$$\det T_{n}^{\left(2\right)}={\sqrt{q}}^{n}{\left(\frac{\sqrt{p_{1}}}{\sqrt{p_{2}}}\right)}^{\delta_{n,\text{odd}}}U_{n}\left(\frac{\sqrt{p_{1}p_{2}}}{2\sqrt{q}}\right)\;,$$

where $\delta_{n,\text{odd}}=1$, if *n* is odd, and 0, otherwise. The general case can be deduced independently from [29], namely, in the following way

$$\det T_{2\ell }^{\left(2\right)}={\left(\sqrt{q_{1}q_{2}}\right)}^{\ell }(U_{\ell }\left(\frac{p_{1}p_{2}-q_{1}-q_{2}}{2\sqrt{q_{1}q_{2}}}\right)+\frac{\sqrt{q_{2}}}{\sqrt{q_{1}}}\;U_{\ell -1}\left(\frac{p_{1}p_{2}-q_{1}-q_{2}}{2\sqrt{q_{1}q_{2}}}\right))$$

and, otherwise,

$$\det T_{2\ell +1}^{\left(2\right)}=p_{1}{\left(\sqrt{q_{1}q_{2}}\right)}^{\ell }\;U_{\ell }\left(\frac{p_{1}p_{2}-q_{1}-q_{2}}{2\sqrt{q_{1}q_{2}}}\right)\;.$$

As in (2.2), with the perturbation of the two first diagonal entries of $T_{n}^{\left(2\right)}$ defined in (4.2), i.e.,

$${\overset{˜}{T}}_{n}^{\left(2\right)}={\left(\begin{array}{ccccccc} p_{1}+r\; & 1\; & \; & \; & \; & \; & \\ q_{1}\; & p_{2}+s\; & 1\; & \; & \; & \; & \\ \; & q_{2}\; & p_{1}\; & 1\; & \; & \; & \\ \; & \; & q_{1}\; & p_{2}\; & 1\; & \; & \\ \; & \; & \; & q_{2}\; & \ddots \; & \ddots \; & \\ \; & \; & \; & \; & \ddots \; & \ddots \; & \ddots \\ \; & \; & \; & \; & \; & \ddots \; & \end{array}\right)}_{n\times n}.$$

We get similar relations (2.3)-(2.4) for the determinant of ${\overset{˜}{T}}_{n}^{\left(2\right)}$. Indeed, assuming a≠0, if we set

$$p_{1}+r=a\;\text{and}\;\left|\begin{array}{cc} a\; & 1\\ q_{1}\; & p_{2}+s \end{array}\right|=b$$

that is, as in (2.5),

(4.3) $$r=a-p_{1}\;\text{and}\;s=\frac{b-ap_{2}+q_{1}}{a}\;,$$

then we obtain

(4.4) $$\det{\overset{˜}{T}}_{n}^{\left(2\right)}=\det T_{n}^{\left(2\right)}+r\det Q_{n-1}^{\left(2\right)}+s(r+p_{1})\det T_{n-2}^{\left(2\right)}\;,$$

where

$$Q_{n}^{\left(2\right)}={\left(\begin{array}{ccccccc} p_{2}\; & 1\; & \; & \; & \; & \; & \\ q_{2}\; & p_{1}\; & 1\; & \; & \; & \; & \\ \; & q_{1}\; & p_{2}\; & 1\; & \; & \; & \\ \; & \; & q_{2}\; & p_{1}\; & 1\; & \; & \\ \; & \; & \; & q_{1}\; & \ddots \; & \ddots \; & \\ \; & \; & \; & \; & \ddots \; & \ddots \; & \ddots \\ \; & \; & \; & \; & \; & \ddots \; & \end{array}\right)}_{n\times n}\;.$$

Finally,

$$w_{n}^{\left(2\right)}=\det{\overset{˜}{T}}_{n+1}^{\left(2\right)}\;,\;\text{for any }n⩾0.$$

Remark 4.1 The formula (4.4) is still true when a=0. For example, the sequence

0,1,1,3,5,13,23,59,105,269,479,1227,2185,5597,…

is known in *The On-Line Encyclopedia of Integer Sequences* [34] as *A*005824. It was considered in [30] by Shallit in the context of the worst-case behavior of three iterative algorithms for computing the Jacobi symbol. It can be defined as

$$f_{n}=\left\{ \begin{array}{cc} 2f_{n-1}+f_{n-2}\; & \text{ if}\;\text{n}\;\text{is odd}\\ f_{n-1}+2f_{n-2}\; & \text{ if}\;\text{n}\;\text{is even} \end{array}\right.,$$

for n⩾2, with initial conditions $f_{0}=0$ and $f_{1}=1$. The determinantal formula for this sequence is

$$f_{n}={\left|\begin{array}{cccccc} 0\; & 1\; & \; & \; & \; & \\ -1\; & 2\; & 1\; & \; & \; & \\ \; & -2\; & 1\; & 1\; & \; & \\ \; & \; & -1\; & 2\; & 1\; & \\ \; & \; & \; & -2\; & 1\; & \ddots \\ \; & \; & \; & \; & \ddots \; & \ddots \end{array}\right|}_{\left(n+1)\times (n+1\right)}={\left|\begin{array}{ccccccc} 0\; & \;1\; & \; & \; & \; & \;\\ -1\; & \;0\; & \; & \; & \; & \; & \\ \; & \;\; & 1\; & 1\; & \; & \; & \\ \; & \;\; & -1\; & 2\; & 1\; & \; & \\ \; & \;\; & \; & -2\; & 1\; & 1\; & \\ \; & \;\; & \; & \; & -1\; & 2\; & \ddots \\ \; & \;\; & \; & \; & \; & \ddots \; & \ddots \end{array}\right|}_{\left(n+1)\times (n+1\right)}.$$

## 5 Examples

In this section we consider two non-trivial cases. The first example is the sequence

(5.1) 1,2,3,7,11,25,39,89,139,317,495,1129,1763,4021,…

coined in *The On-Line Encyclopedia of Integer Sequences* as *A*007481. This sequence lists the number of subsequences of {1,2,…,n} in which every even member has at least one odd neighbor, for each n⩾0. The empty sequence is acceptable. A similar sequence has been defined by Guy and Moser [20] where the roles of odd and even are interchanged. This one is *A*007455 in OEIS.

The sequence (5.1) satisfies the recurrence relation

$$z_{n}=3z_{n-2}+2z_{n-4}\;.$$

As far as we can tell, an explicit formula is not known. Now, if we consider the sequence $\left\{f_{n}\right\}$ defined by the recurrence

$$f_{n}=\left\{ \begin{array}{cc} 2f_{n-1}-f_{n-2}\; & \text{ if}\;\text{n}\;\text{is even}\\ f_{n-1}+2f_{n-2}\; & \text{ if}\;\text{n}\;\text{is odd} \end{array}\right.$$

for n⩾2, with initial conditions $f_{0}=1$ and $f_{1}=2$, it turns out that $z_{n}=f_{n}$, for any nonnegative integer *n*. From (4.3) we have

$$f_{n}={\left|\begin{array}{ccccccc} 1\; & 1\; & \; & \; & \; & \;\\ -2\; & 0\; & 1\; & \; & \; & \; & \\ \; & 1\; & 2\; & 1\; & \; & \; & \\ \; & \; & -2\; & 1\; & 1\; & \; & \\ \; & \; & \; & 1\; & 2\; & 1\; & \\ \; & \; & \; & \; & -2\; & 1\; & \ddots \\ \; & \; & \; & \; & \; & \ddots \; & \ddots \end{array}\right|}_{\left(n+1)\times (n+1\right)}.$$

Since r=s=−1, from (4.4), we may also write

$$f_{2n}={\left(-i\sqrt{2}\right)}^{n}U_{n}\left(\frac{3i\sqrt{2}}{4}\right)$$

and

$$f_{2n+1}={\left(-i\sqrt{2}\right)}^{n}\left(2\;U_{n}\left(\frac{3i\sqrt{2}}{4}\right)+\frac{i\sqrt{2}}{2}\;U_{n-1}\left(\frac{3i\sqrt{2}}{4}\right)\right)\;,$$

for n⩾0.

The second sequence that we would like to consider is

(5.2) 1,2,3,5,7,12,17,29,41,70,99,169,239,408,577,…

which is *A*002965 in OEIS. This sequence is not only a mere mathematical object. In fact, it goes far beyond it, as one can find it in the *Battistero di San Giovanni* in Florence [37]. The sequence (5.2) satisfies the recurrence

$$g_{n}=\left\{ \begin{array}{cc} 2g_{n-1}-g_{n-2}\; & \text{ if}\;\text{n}\;\text{is even}\\ g_{n-1}+g_{n-2}\; & \text{ if}\;\text{n}\;\text{is odd} \end{array}\right.$$

for n⩾2, with initial conditions $g_{0}=1$ and $g_{1}=2$. This means that the explicit form for this sequence is

$$g_{2n}={\left(-i\right)}^{n}\left(U_{n}\left(i\right)+i\;U_{n-1}\left(i\right)\right)$$

and

$$g_{2n+1}={\left(-i\right)}^{n}\left(2\;U_{n}\left(i\right)+i\;U_{n-1}\left(i\right)\right)\;,$$

for n⩾0, while in terms of a tridiagonal matrix we have

$$g_{n}={\left|\begin{array}{ccccccc} 1\; & 1\; & \; & \; & \; & \;\\ -1\; & 1\; & 1\; & \; & \; & \; & \\ \; & 1\; & 2\; & 1\; & \; & \; & \\ \; & \; & -1\; & 1\; & 1\; & \; & \\ \; & \; & \; & 1\; & 2\; & 1\; & \\ \; & \; & \; & \; & -1\; & 2\; & \ddots \\ \; & \; & \; & \; & \; & \ddots \; & \ddots \end{array}\right|}_{\left(n+1)\times (n+1\right)}.$$

## Declarations

### Author contribution statement

Fatih Yilmaz, Milica Anđelić, Carlos M. da Fonseca: Conceived and designed the experiments; Performed the experiments; Analyzed and interpreted the data; Wrote the paper.

### Funding statement

This research did not receive any specific grant from funding agencies in the public, commercial, or not-for-profit sectors.

### Data availability statement

No data was used for the research described in the article.

### Declaration of interests statement

The authors declare no conflict of interest.

### Additional information

No additional information is available for this paper.
